# Overexpression of aquaporin-1 plays a vital role in proliferation, apoptosis, and pyroptosis of Wilms’ tumor cells

**DOI:** 10.1007/s00432-024-05616-6

**Published:** 2024-02-09

**Authors:** Hong Liu, Chen Jin, Nan Xia, Qian Dong

**Affiliations:** Qingdao University, 16 Jiangsu Road, Qingdao, 266000 Shandong China

**Keywords:** Wilms’ tumor, Aquaporin-1, Proliferation, Apoptosis, Pyroptosis

## Abstract

**Background:**

Nephroblastoma, also known as Wilms’ tumor (WT), is an embryonic malignant tumor and one of the most common malignant tumors in the abdominal region of children. The exact role and underlying mechanisms of aquaporin-1 (AQP1) in the occurrence and development of nephroblastoma remain unclear.

**Methods:**

After overexpression of AQP1, cell proliferation was assessed using the CCK-8 proliferation assay and EdU staining. Flow cytometry was employed to assess cell apoptosis, and Western blotting (WB) analysis was conducted to validate the expression of relevant protein markers. mRNA sequencing (mRNA-Seq) was performed on WT cells overexpressing AQP1 to predict and characterize the associated mechanisms. Transmission electron microscopy was utilized to observe changes in the ultrastructure of WT cells undergoing apoptosis and pyroptosis following AQP1 overexpression. Functional in vivo validation was conducted through animal experiments.

**Results:**

We validated that overexpression of AQP1 inhibited cell proliferation and promoted cell apoptosis and pyroptosis both in vitro and in vivo. mRNA-Seq analysis of WT cells with AQP1 overexpression suggested that these effects might be mediated through the inhibition of the JAK-STAT signaling pathway. Additionally, we discovered that overexpression of AQP1 activated the classical pyroptosis signaling pathway dependent on caspase-1, thereby promoting pyroptosis in WT.

**Conclusion:**

These findings highlight the important functional role of AQP1 in the pathobiology of nephroblastoma, providing novel insights into the development of this disease. Moreover, these results offer new perspectives on the potential therapeutic targeting of AQP1 as a treatment strategy for nephroblastoma.

## Introduction

Wilms’ tumor (WT) accounts for approximately 6% of all childhood cancers and is the most common malignant renal tumor in children. Currently, the diagnosis of WT primarily relies on clinical manifestations and imaging examinations, such as CT scans. Although CT exhibits high sensitivity in diagnosing WT, the specificity is low, resulting in a low rate of early detection. Furthermore, while a combination of surgery and chemotherapy has proven effective in achieving favorable treatment outcomes for the majority of WT patients, some children with high-risk histology still show resistance to conventional therapies, leading to a higher rate of postoperative recurrence. Currently, many studies have focused on identifying the mechanisms underlying the occurrence and development of WT. The factors involved in WT pathogenesis are exceedingly complex, with an intricate interplay among them, and remain unclear. Therefore, researchers need to elucidate the etiology of WT to address the challenges associated with the early diagnosis, early treatment, and prognosis of WT. As the first discovered water channel protein (Benga 1988), aquaporin-1 (AQP1) primarily functions by regulating the rapid movement of water across the plasma membrane, thereby influencing the osmotic gradient between the intracellular and extracellular environments (Moon et al. 1993). Studies have found that urinary AQP1 can serve as a potential biomarker for clear-cell renal cell carcinoma (ccRCC) (Mijuskovic et al. 2016). Northern blot analysis showed that the high expression of AQP1 is associated with a good prognosis in patients with ccRCC (Takenawa et al. 1998). An immunohistochemistry (IHC) assay indicated that AQP1 is a potential expression marker for low-grade ccRCC (Mazal et al. 2005). Huang et al. discovered that the expression of AQP1 is significantly negatively correlated with important clinical parameters of RCC, such as tumor size, stage, and symptoms, as well as important histological parameters, such as microvascular invasion. The expression of AQP1 was also significantly negatively correlated with tumor grade and specific survival in ccRCC. The authors proposed that AQP1 could serve as a significant prognostic indicator for ccRCC (Huang et al. 2009). In our preliminary study, we conducted bioinformatics analysis and identified AQP1 as a potential key tumor suppressor gene involved in the occurrence and development of WT (Liu et al. 2023). However, the impact of AQP1 expression changes on the functionality of WT and the underlying mechanisms remain unclear. Moreover, pyroptosis is a form of programmed cell death characterized by its lytic and inflammatory nature. Studies have shown that pyroptosis in tumors can induce cytokine release, promoting tumor occurrence, and infiltration (Balkwill and Mantovani 2001), while also playing a crucial role in antitumor immune function and inhibiting tumor growth (Wei et al. 2022). Research has demonstrated the close association of AQP2 (Lyu et al. 2021) and AQP4 (Al-Dhohorah et al. 2016) from the AQP family with pyroptosis. However, the involvement of AQP1 in pyroptosis in WT and its related mechanisms have yet to be verified.

In this study, we validated the impact of AQP1 overexpression on cell proliferation, apoptosis, and pyroptosis in WT cells through in vitro and in vivo experiments. We investigated the functional changes and potential mechanisms underlying the role of AQP1 as a potential biomarker in the occurrence and development of WT.

## Materials and methods

### Materials

We chose two WT cell lines, SK-NEP-1 and WiT-49.

SK-NEP-1 cells were from the National Collection of Authenticated Cell Cultures, and WiT-49 cells were from the Shanghai Cell Bank. Fetal bovine serum (FBS), Dulbecco’s modified Eagle’s medium (DMEM), and Opti-Minimum Essential Medium (Opti-MEM) were obtained from BI Corporation (MD, USA). Radioimmunoprecipitation assay lysis buffer (RIPA), sodium dodecyl sulfate‒polyacrylamide gel electrophoresis (SDS‒PAGE) gels, a bicinchoninic acid (BCA) protein quantification kit, and electrochemiluminescence (ECL) reagents were purchased from Beyotime Biotechnology Co., Ltd. (Beijing, China). TRIzol Reagent was purchased from Invitrogen (CA, USA). AQP1, β-actin, GAPDH, c-Myc, BCL-2, cleaved caspase-3, cleaved caspase-1, GADMD, NLRP3, JAK2, and STAT3 antibodies were all obtained from Abcam (Cambridge, UK).

Cleaved IL-1β, P-JAK2, and P-STAT3 antibodies were obtained from CST (MA, USA). PYCARD (ASC) and P-STAT1 antibodies were purchased from eLife Company (Guangzhou, China). The goat anti-rabbit IgG antibody was purchased from Jackson (MC, USA), and the goat anti-mouse IgG antibody was purchased from Santa Cruz (California, USA). The CCK-8 assay kit was purchased from Biosharp (Guangzhou, China). The EdU assay kit was purchased from Cellorlab (Shanghai, China). The Annexin V-APC/7-AAD apoptosis kit was purchased from Lianke Biotech Company (Hangzhou, China). The AQP1 overexpression lentivirus was acquired from Genechem Biotech Company (Shanghai, China). mRNA sequencing (mRNA-Seq) was performed by Biomarker Technological Co., Ltd. (Beijing, China). BALB/c nude mice were purchased from Beijing Vital River Laboratory Animal Technology Co., Ltd. (Beijing, China). All the animal care and processes involved in this experiment were enforced in conformity with the Animal Welfare and Research Ethics Committee at Qingdao University (Approval ID: 20220107BALB/c Nude1620220525085).

### Methods

#### Establishment of stable lentivirus-transduced cell lines with AQP1 overexpression

The coding sequence of the AQP1 gene was derived from the AQP1 gene sequence (NM_198098) provided by GenBank. The primer pairs were as follows: AQP1 forward: 5′-GCTATGCGTGCTGGCTACTA-3′, AQP1 reverse: 5′-TCCCACAGCCAGTGTAGTCA-3′. GAPDH was used as a reference for each sample.

The stable lentivirus-transduced AQP1 overexpression group and negative control group of SK-NEP-1 and WiT-49 cell lines were generated by puromycin selection at a concentration of 1 μg/ml. Both negative control and overexpression AQP1 cell lines were cultured in DMEM with 10% FBS, 1% penicillin, and 1% streptomycin. The cell lines were cultured in a standard cell culture incubator at 37 °C with 5% CO_2_.

#### CCK-8 assay for cell proliferation

Cells in the negative control group and overexpression AQP1 group were resuspended and plated in 96-well plates at a seeding density of 4 × 10^3^ cells per well. The cells were incubated in each well at 37 °C for the designated time points: 0 h, 24 h, 48 h, 72 h, and 96 h. After each incubation period, the old culture medium was discarded and replaced with 100 μl of complete DMEM containing 10% FBS, and then, 10 µl of CCK-8 detection solution was added to each well. After the cells were incubated for 2 h, the optical density (OD) at 450 nm was measured by a microplate reader. Three replicate wells were set up for each group, and the cell proliferative capacity for each group was calculated.

#### EdU assay for cell proliferation

SK-NEP-1 and WiT-49 cells from the negative control group and AQP1 overexpression group were plated in 96-well plates at a seeding density of 6 × 10^3^ cells per well. Then, 100 μl of EdU solution (diluted in DMEM at a ratio of 1000:1) was added to each well and incubated at 37 °C for 2 h. The cells were fixed and stained according to the instructions of the EdU assay kit. Five fields of view were randomly selected under a microscope, and the number of positive cells and the cell proliferation rate were calculated at different time points. Cell proliferation rate = (number of positive cells/total number of cells) × 100%.

### Apoptosis analysis by flow cytometry

Each group of cells was digested using trypsin, collected, and resuspended in 500 μl of binding buffer. Then, 10 μl of Annexin V-APC and 5 μl of 7-AAD were added to the cell suspension, mixed gently, and incubated at 4 °C in the dark for 15 min. Cell apoptosis was detected using a flow cytometer.

### Tumor xenografts in BALB/c nude mice

Twelve 5-week-old male BALB/c nude mice were selected and housed in a specific pathogen-free (SPF) environment. They were randomly divided into two groups: the control group and the AQP1 overexpression group, with six mice in each group. The mice were fed a standard laboratory diet. Once the nude mice reached a stable condition, they were subcutaneously inoculated in the axillary region with empty vector cells and AQP1-overexpressing WiT-49 stably transduced cells, each at a dose of 2 × 10^7^ cells. The weight of the nude mice and the tumor volume were measured once a week. The tumor volume was calculated using the formula: tumor volume = (length × width^2^)/2. The nude mice were euthanized after 8 weeks by cervical dislocation. Tumors from each group were collected and weighed. Tumor tissues were dissected for further analysis. The specimens were used for imaging, Western blotting (WB) and IHC.

### RNA sequencing analyses

Total RNA extraction from each sample was performed following the instructions provided in the TRIzol reagent manual. Subsequently, RNA qualification, library construction and sequencing were conducted by Beijing Biomarker Technologies Co., Ltd. The threshold for identifying the significance of the *P* value in multiple experiments was determined using the false discovery rate (FDR) control method. Further analysis was conducted on the differentially expressed genes (DEGs) based on the criteria of fold change (FC) ≥ 1.2 and *P* value < 0.05.

### WB

The proteins were extracted using RIPA lysis buffer, and the protein concentrations were determined using the BCA assay. Subsequently, 30 μg of protein per well was separated by 10% SDS‒PAGE. The separated proteins were then transferred onto a PVDF membrane. The membrane was blocked at room temperature using a blocking solution containing 5% skim milk powder. The appropriate monoclonal antibodies were added at a dilution of 1:1000 and incubated overnight at 4 °C. Monoclonal antibodies against β-actin or GAPDH were used as internal references at a dilution of 1:2000. The next day, the PVDF membrane was washed three times with TBST. HRP-conjugated secondary antibodies (1:10,000) were added and incubated at room temperature for 1 h. After the membrane was washed with Tris-buffered saline with Tween 20 (TBST), ECL reagent was used for chemiluminescent detection. The luminescent signals were detected using the AlphaView imaging system from FluorChemQ.

### IHC

Prior to staining, the paraffin-embedded tissue specimen was sectioned into 4 μm slices, followed by dewaxing and rinsing in anhydrous xylene. For antigen retrieval, the sections were subsequently rehydrated using a gradient of ethanol. Afterward, the sliced samples were exposed to 3% hydrogen peroxide to inhibit endogenous peroxidase activity. Subsequently, the samples were washed three times with phosphate-buffered saline (PBS) and then sealed with a 5% FBS–PBS solution for 15 min. Finally, the sections were incubated overnight at 4 °C with the designated antibody. The following day, the sections were subjected to a 60-min incubation with the secondary antibody. Subsequently, immunohistochemical avidin–biotin complex (ABC) staining was employed to detect the presence of 3,3′-diaminobenzidine (DAB). Positive staining was observed as a yellow or brown coloration in the cytoplasm or nucleus when viewed under a light microscope, while unstained samples were considered negative. The immunohistochemical photos were captured at a magnification of 200×. ImageJ software was utilized for the analysis of optical density. For determination of the cumulative integrated optical density (IOD), three pathological sections were selected from each specimen, and the average value was calculated.

### Transmission electron microscopy

The cells of the different groups were digested using 0.25% trypsin. Subsequently, the cells were fixed with 1.5% glutaraldehyde at 4 °C for 6 h. Ultrathin sections (100 nm) were obtained and stained with uranyl acetate and lead citrate. The sections were then examined under a transmission electron microscope (TEM) (H-600; Hitachi, Tokyo, Japan).

### Statistical analyses

Data processing was performed using SPSS 26.0 software. Experimental data are presented as the mean ± standard deviation (*x* ± *s*). Each experiment was independently repeated three times. Data comparisons between two groups were performed with a *t* test, while comparisons among multiple groups were performed with one-way analysis of variance (ANOVA). *P* < 0.05 was considered significant.

## Results

### Overexpression of AQP1 inhibits proliferation of WT cells

**Fig. 1:**
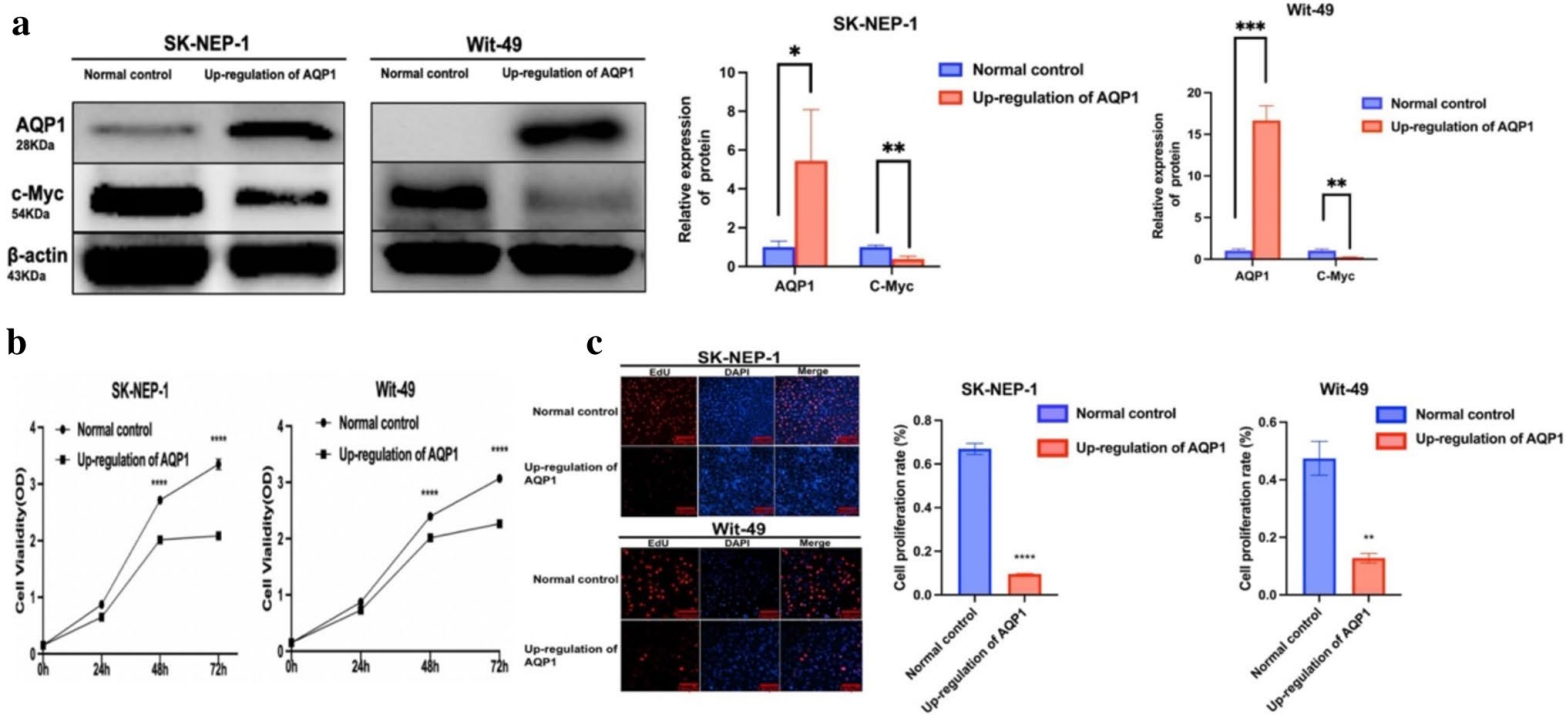
Up-regulation of AQP1 suppressed Wilms’ tumor cell proliferation. **a** Stable cell lines were generated using lentiviral overexpression AQP1 system. The up-regulation efficiency was measured by Western blotting. The expression of c-Myc in SK-NEP-1 and Wit-49 cells were detected by Western blotting (WB). Relative expression of proteins. Data are presented as mean ± SD. Statistic significant differences were indicated: *P < 0.05, **P < 0.01, ***P < 0.001. **b** CCK-8 assay after AQP1 overexpression in SK-NEP-1 and Wit-49 cells (*n* = 3). Data are presented as mean ± SD. Statistic significant differences were indicated: ****P < 0.0001. **c** The cell proliferation was examined by EdU assays (magnification, ×200). EdU-positive cell rate. Data are presented as mean ± SD. Statistic significant differences were indicated: ***P < 0.001, ****P < 0.0001

A functional investigation was conducted on the control group and the AQP1 overexpression group. The transfection efficiency of AQP1 overexpression was evaluated via WB, while the expression levels of the proliferation-associated biomarker c-Myc were examined in both the AQP1 overexpression group and the control group of WT cells. We observed a significant decrease in the expression of c-Myc in the AQP1 overexpression group compared to the control group (Fig. 1a). To further determine the effect of AQP1 overexpression in cell proliferation, we conducted CCK-8 experiments. The results revealed a significant decrease in the proliferative capacity of SK-NEP-1 and Wit-49 cell lines in the AQP1 overexpression group (*P* < 0.0001) (Fig. 1b). Additionally, an EdU assay demonstrated a significant reduction in the proliferative capacity of the AQP1 overexpression group compared to the control group (Fig. 1c). The difference between the AQP1 overexpression group and the control group was statistically significant (*P* < 0.0001, *P* < 0.01).

### Overexpression of AQP1 promotes apoptosis of WT cells

**Fig. 2:**
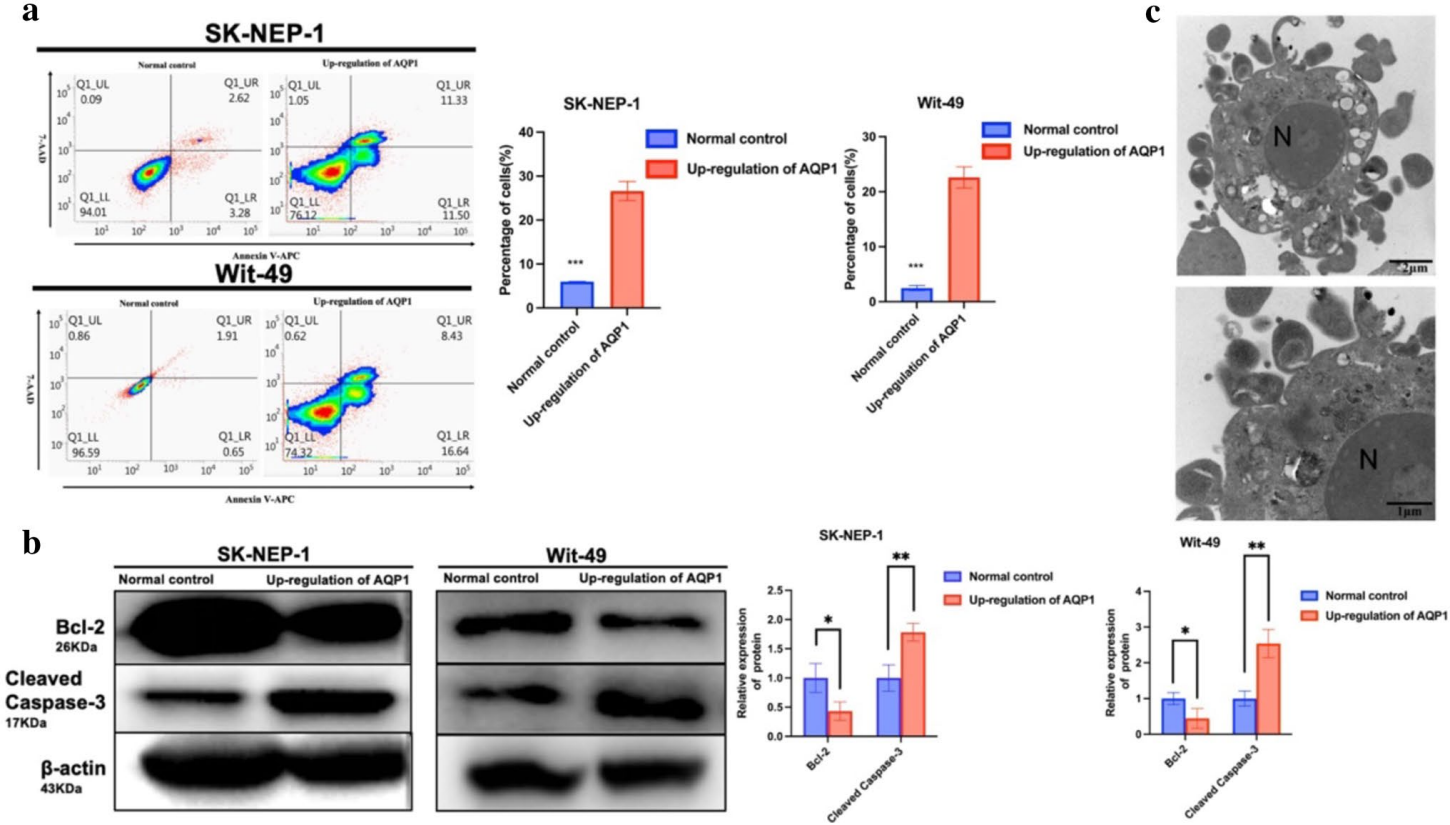
Up-regulation of AQP1 promoted apoptosis. **a** Apoptosis analyzed by flow cytometry showing the increased apoptosis of SK-NEP-1 and Wit-49 cells after up-regulation of AQP1. The apoptosis rate of SK-NEP-1 and Wit-49 cells. Data are presented as mean ± SD (*n* = 3). Statistic significant differences were indicated: ***P < 0.001. **b** WB analysis of BCL-2, Cleaved Caspase-3 in SK-NEP-1 and Wit-49 cells, with up-regulation of AQP1 and normal control. Relative expression of proteins. Data are presented as mean ± SD. Statistic significant differences were indicated: *P < 0.05, **P < 0.01. **c** The apoptosis in up-regulation of AQP1 group were observed by transmission electron microscopy (magnification, × 15,000). At higher magnification, up-regulation of AQP1 cells were characterized by prominent membrane blebs (black arrows) while maintaining apparent cytoplasmic and nuclear organization (magnification, × 30,000). *N* nucleus

To determine the role of AQP1 in apoptosis of WT cells, we utilized flow cytometry to detect apoptosis. The results showed that the apoptotic rate of the AQP1 overexpression group was significantly higher than that of the control group (Fig. 2a). The difference between the two groups was statistically significant (*P* < 0.001). We also examined the expression levels of the apoptosis-related biomarkers BCL-2 and cleaved caspase-3 using WB. Compared to those in the control group, the expression levels of BCL-2 and cleaved caspase-3 were significantly decreased in the AQP1 overexpression group (Fig. 2b). The above results exhibited statistically significant differences between the two groups. Furthermore, we explored the ultrastructure of apoptosis using a TEM. In the AQP1 overexpression group, there was a notable increase in typical apoptotic bodies, characterized by reduced cell volume, cytoplasmic condensation, chromatin aggregation, intact nuclear membranes, plasma membranes, and preserved organelles. The cell membrane exhibited budding and then the formation of apoptotic bodies (Fig. 2c). The results indicate that overexpression of AQP1 promotes apoptosis in WT.

### Overexpression of AQP1 inhibits the growth of WT cells in vivo

**Fig. 3:**
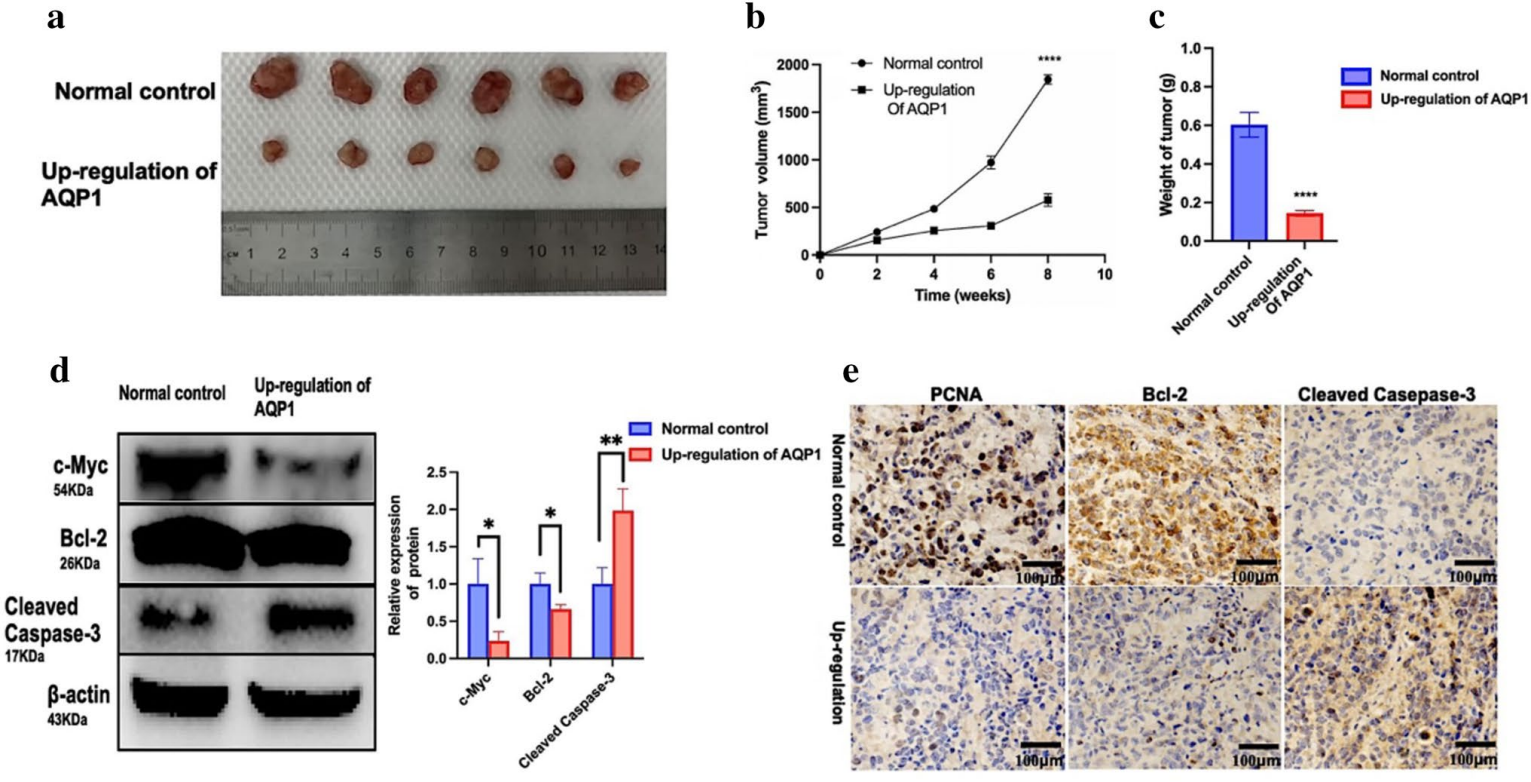
Inhibition of WT development in nude mice after up-regulation of AQP1. **a** WT in nude mice. Stable Wit-49 cell lines after up-regulation of AQP1 and control Wit-49 cells were harvested, counted, and suspended in an equal volume of high-concentrated Matrigel and then 100 µl of the suspension (5 × 10^6^ cells) was injected under the axilla skin of nude mice. Photograph and comparison of excised tumor size. **b** The changes of tumor volume of subcutaneous tumor-bearing nude mice at the indicated time. Mean ± SD, *n* = 6, **P < 0.01. **c** Tumor weight at the experimental end point (8th week). Mean ± SD, *n* = 6, ****P < 0.0001. **d** WB analysis of c-Myc, BCL-2, and Cleaved Caspase-3 in up-regulation of AQP1 group and normal control group. Relative expression of proteins. Data are presented as mean ± SD. Statistic significant differences were indicated: *P < 0.05, **P < 0.01. **e** Levels of PCNA, BCL-2, and Cleaved Caspase-3 were detected by immunohistochemistry (IHC) (magnification, × 200)

All nude mice were successfully xenografted, and gross specimen images of the obtained xenografted tumors are described in Fig. 3a. The measurement results of tumor weight and volume are shown in Fig. 3b, c, respectively. Compared to those in the control group, the weight and average volume of tumors in the AQP1 overexpression group were significantly reduced (*P* < 0.0001). WB analysis (Fig. 3d) was performed to detect the expression of c-Myc, BCL-2, and cleaved caspase-3 in tumors. Compared to the control group, the AQP1 overexpression group showed decreased expression levels of c-Myc and BCL-2, while the expression levels of cleaved caspase-3 were increased in tumors. This finding indicates that AQP1 overexpression inhibits proliferation and promotes apoptosis in WT cells. IHC (Fig. 3e) was used to assess the expression of PCNA, BCL-2, and cleaved caspase-3 in tumor tissues, revealing decreased expression of PCNA and BCL-2 and increased expression of cleaved caspase-3. The above results exhibited significant differences between the two groups. The results suggest that overexpression of AQP1 can inhibit proliferation and promote apoptosis of tumors in vivo.

In summary, the overexpression of AQP1 in nude mice inhibited WT growth.

### Potential mechanisms underlying the overexpression of AQP1

**Fig. 4:**
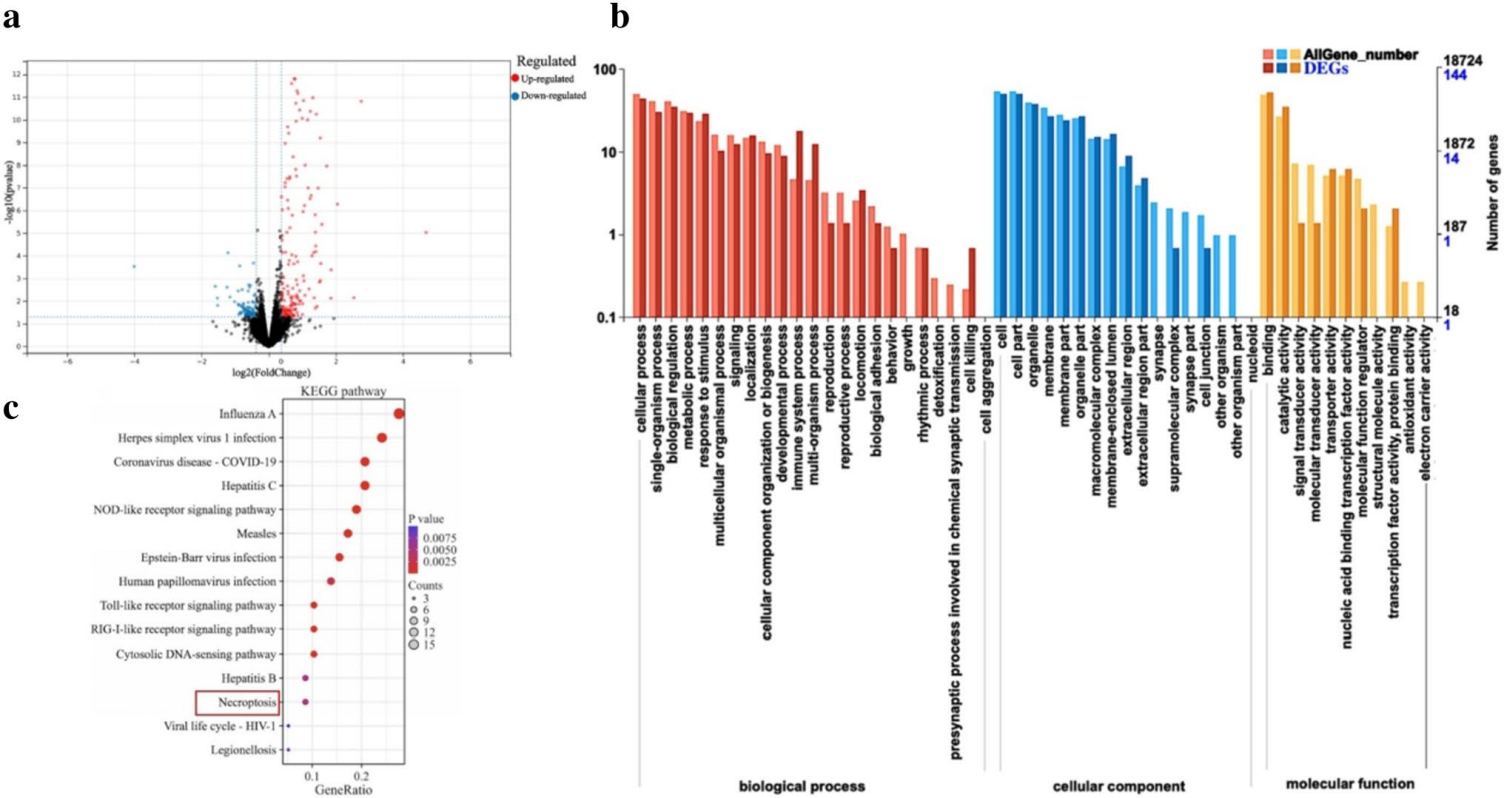
mRNA sequencing (mRNA-seq) reads from up-regulation of AQP1 and control Wit-49 cells. **a** Volcano plot of differentially expressed genes (DEGs) was shown of up-regulation of AQP1 and control Wit-49 cells. **b** The GO enrichment analysis of DEGs. **c** KEGG pathway analysis of DEGs

mRNA-Seq was utilized to analyze the potential mechanism of AQP1 overexpression in Wit-49 cells and control Wit-49 cells. A volcano plot of differentially expressed genes is shown in Fig. 4a. The results revealed the identification of 47 DEGs in the AQP1-overexpressing Wit-49 cells: 38 upregulated genes and 9 downregulated genes [|log2-fold change (FC)|≥ 1.2 and *P* value < 0.05]. The GO enrichment analysis included categories such as biological process (BP), cell component (CC), and molecular function (MF) of the DEGs (Fig. 4b). We conducted an analysis of the genes with the strongest upregulated or downregulated expression among the DEGs and found that many genes exhibit functions closely associated with cell proliferation, apoptosis, and immune and inflammatory regulation, which are closely related to pyroptosis (Table 1). The KEGG pathway analysis is depicted in Fig. 4c. Based on the KEGG pathway analysis of DEGs, we identified necroptosis as one of the most significantly enriched pathways. This finding is consistent with our previous validation that overexpression of AQP1 promotes apoptosis in WT. The DEGs associated with necroptosis included caspase-1, STAT1, PYCARD (ASC), TLR3, and JMJD7-PLA2G4B. Based on this, we hypothesized that the overexpression of AQP1 may affect the JAK–STAT signaling pathway, thereby affecting cell proliferation and apoptosis. In addition, caspase-1 and PYCARD (ASC) are key marker proteins in the classic pathway of pyroptosis. We also further validated whether overexpression of AQP1 affects pyroptosis.

**Table 1:** Most relevant genes that displayed the greatest changes in their expressions in the overexpression of AQP1 WT cells

Gene symbol	Name	Fold change	Biological function
RSAD2	Radical *S*-adenosyl methionine domain-containing protein 2	2.75590748	In addition to its antiviral and immune regulatory functions, it has been found to be associated with cellular proliferation, cell cycle regulation, cell migration, and apoptosis
CMTM1	CKLF-like MARVEL transmembrane domain-containing 1	2.5357084	It interacts with immune regulatory cytokines and receptors, exhibits tumor-suppressive effects, inhibits tumor cell proliferation, invasion, and metastasis, and induces tumor cell apoptosis
LAMP3	Lysosomal-associated membrane protein 3	1.86007351	It participates in immune regulation and regulates biological behaviors of tumor cells, including proliferation, apoptosis, invasion, and metastasis
CDRT4	Cell division cycle-related and testis-specific 4	1.85455022	It is involved in regulating cell division and cell cycle progression. The cell cycle is a fundamental process of cell growth and reproduction
SLC15A3	Solute carrier family 15 member 3	1.72855877	It is a peptide transporter involved in the transport and translocation of peptide substances
KLHDC7B	Kelch domain containing 7B	1.58266478	It is a protein containing Kelch domain
IFIT2	Interferon-induced protein with tetratricopeptide repeats 2	1.55571373	IFIT2 is one of the interferon-induced antiviral effect proteins and plays an important role in immune regulation. It is also involved in the regulation of cell cycle and proliferation
C1GALT1C1L	C1 galactosyltransferase 1C1-Like	1.55109332	It is associated with specific glycosyltransferases involved in the glycosylation process
OAS2	2′-5′-oligoadenylate synthetase 2	1.54354349	It enhances the antiviral immune response, strengthens innate immune response, and promotes resistance against pathogen infections
CXCL11	C-X-C motif chemokine ligand 11	1.53769819	It plays a crucial role in immune and inflammatory regulation and is also important in tumor immunity. It can enhance the infiltration of T cells and NK cells and promote immune clearance of tumors
XAF1	XIAP-associated factor 1	1.53663253	XAF1 is considered an important tumor suppressor gene. It can bind to X-linked Inhibitor of Apoptosis Protein (XIAP) and prevent XIAP from inhibiting apoptosis. It promotes the activation of caspases, leading to cell apoptosis. It is also involved in regulating immune responses
CMPK2	Cytidine monophosphate kinase 2	1.51141554	It plays a crucial role in cellular nucleotide metabolism and nucleic acid synthesis
TOMM6	Translocase of outer mitochondrial membrane 6	− 1.5910079	It plays a crucial role in the functionality and metabolic processes of mitochondria, and mitochondria play a key role in cellular apoptosis
BEST1	Bestrophin 1	− 4.0058237	Regulating the balance of intracellular and extracellular ion concentrations

### Overexpression of AQP1 inhibits the activation of the JAK–STAT signaling pathway

**Fig. 5:**
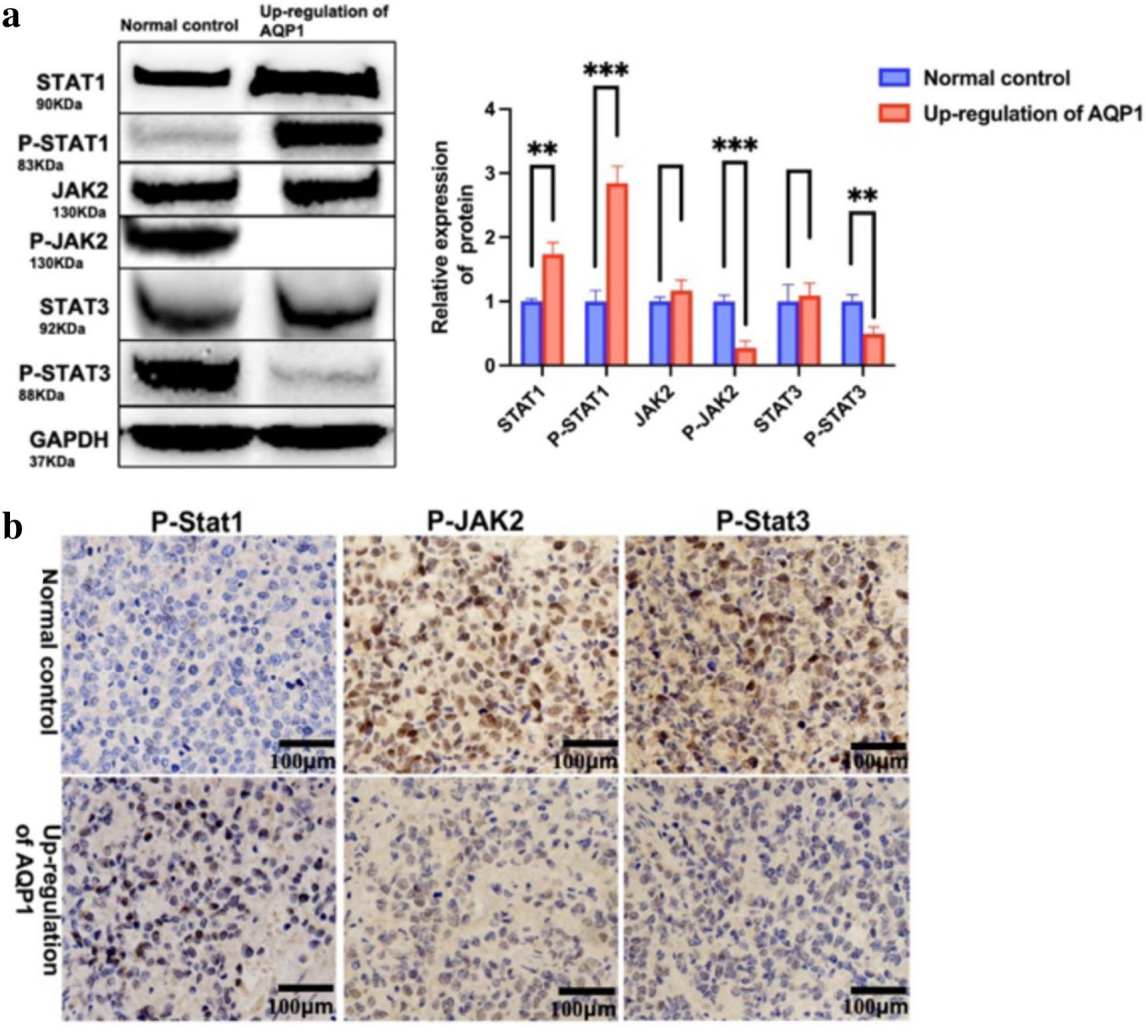
Inhibition of JAK-STAT signaling pathway after up-regulation of AQP1. **a** WB analysis of STAT1, P-STAT1, JAK2, P-JAK2, STAT3, and P-STAT3 in up-regulation of AQP1 group and normal control group. Relative expression of proteins. Data are presented as mean ± SD. Statistic significant differences were indicated: **P < 0.01, ***P < 0.001. **b** Levels of P-STAT1, P-JAK2, and P-STAT3 were detected by IHC in nude mice tumors ( magnification, × 200)

To further validate the potential mechanism of the impact of AQP1 overexpression on proliferation and apoptosis, we performed verification of the marker proteins in the JAK–STAT signaling pathway. The WB results, as depicted in Fig. 5a, revealed that the expression of P-STAT1 increased with AQP1 overexpression in WT cells, which is consistent with the mRNA-Seq results. Furthermore, the expression of P-JAK2 and P-STAT3 decreased with AQP1 overexpression (Fig. 5b). IHC of tumor tissues from nude mice was further utilized for validation. The results demonstrated an increase in P-STAT1 expression, while P-JAK2 and P-STAT3 expression decreased. The above results exhibited statistically significant differences between the two groups. Overall, these findings suggested that overexpression of AQP1 in WT may impact proliferation and apoptosis by inhibiting the JAK-STAT signaling pathway.

### Overexpression of AQP1 promotes pyroptosis in WT

**Fig. 6:**
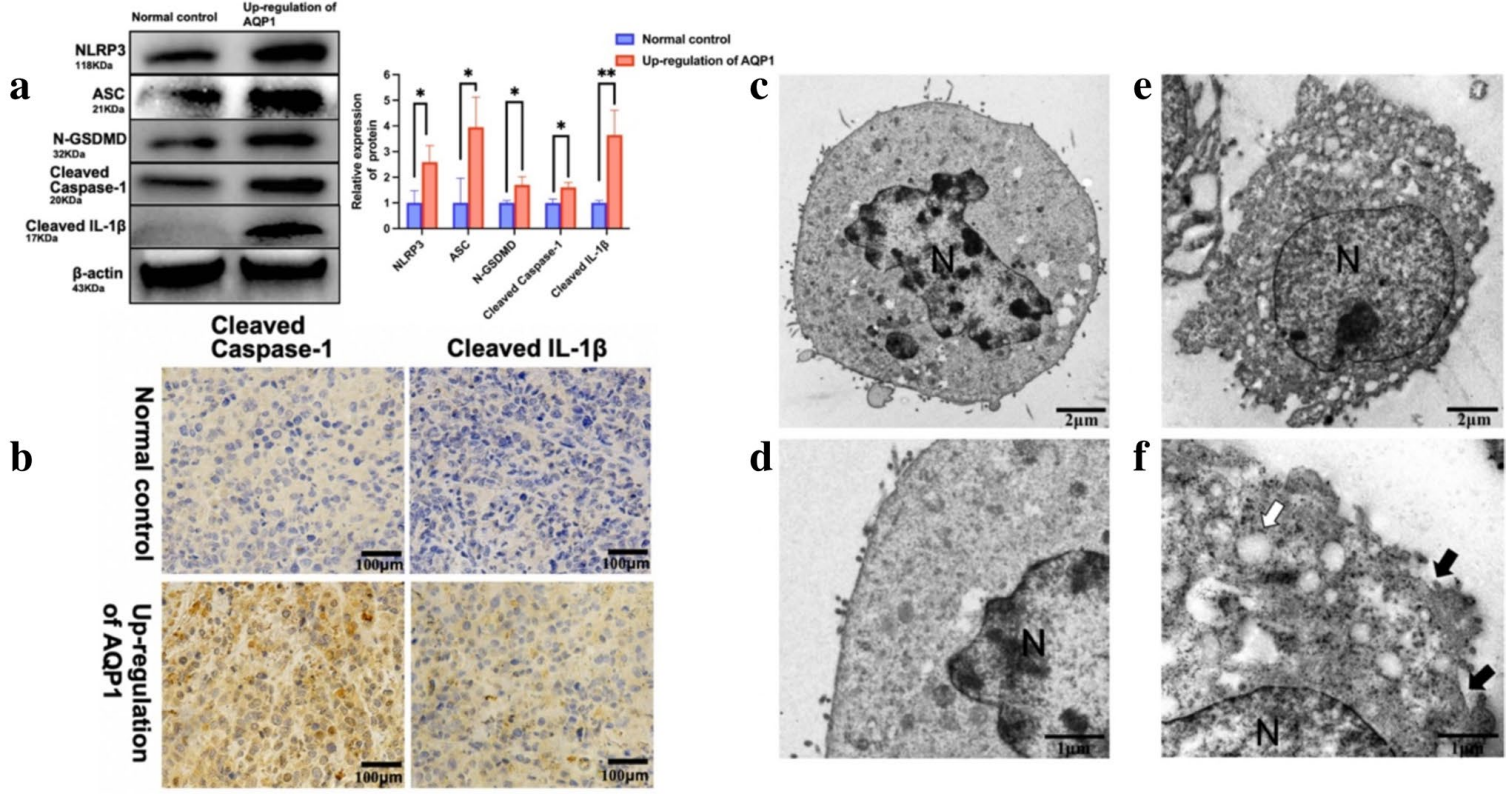
Potential mechanisms that underlie the up-regulation of AQP1 associations with pyroptosis. **a** WB analysis of NLRP3, ASC, N-GSDMD, Cleaved Caspase-1, and Cleaved IL-1β in up-regulation of AQP1 group and normal control group. Relative expression of proteins. Data are presented as mean ± SD. Statistic significant differences were indicated: *P < 0.05, **P < 0.01. **b** Levels of Caspase-1 and Cleaved IL-1β were detected by IHC (magnification, × 200). **c** Representative TEM images present the ultrastructures in normal control group and up-regulation of AQP1 group. The image shows more intact cellular morphologies of WT observed in normal control group. The typical WT cells surface is smooth and the cell membrane is intact. The ratio of nuclei to cytoplasm is significantly increased and the organelles are poorly developed (magnification, × 8000). **d** At higher magnification, WT cells have intact plasma membranes, organelles are underdeveloped (magnification, × 25,000). **e** The image shown more pyroptosis observed in up-regulation of AQP1 group (magnification, × 8000). **f** At higher magnification, up-regulation of AQP1 cells exhibit loss of cytoplasmic, observe a large number of vacuolar structures in the cytoplasm, loss of cell processes (white arrow), and disruptions of the plasma membrane (black arrows), (magnification, × 25,000). *N* nucleus

mRNA-Seq indicated a potential interaction between overexpressed AQP1 and caspase-1 and PYCARD (ASC). To determine whether overexpression of AQP1 is involved in pyroptosis, we validated the expression of key marker proteins in the classic pyroptosis pathway. As shown in Fig. 6a, we confirmed that overexpression of AQP1 in WT cells leads to increased expression of NLRP3, PYCARD (ASC), N-GSDMD, cleaved caspase-1, and cleaved IL-1β. This prediction was further validated through IHC of tumor tissues, as depicted in Fig. 6b, where overexpression of AQP1 resulted in increased expression of cleaved caspase-1 and cleaved IL-1β. These results showed significant differences between the two groups. In addition, as shown in Fig. 6c–f, TEM was utilized to explore the ultrastructure during pyroptosis. Overexpression of AQP1 resulted in the presence of more typical pyroptotic bodies, characterized by cell swelling, incomplete cell membranes, membrane pores on the cell surface, the absence of distinct organelles within the cytoplasm, and the appearance of multiple vacuolar structures. In summary, our results demonstrated that overexpression of AQP1 activates the classic pathway of cellular pyroptosis.

## Discussion

AQP1 is expressed in almost all cell types, and its role in modulating cellular functional changes has been reported in various organs, including the kidney, myocardium, brain, and lungs, as well as in several tumors (Kageyama et al. 1996; Morrissey et al. 2015). However, its specific role in WT remains unclear. In our previous study, we conducted bioinformatics analysis and experimental validation, which revealed significant differential expression of AQP1 in WT compared to normal kidney tissue. This finding suggests that AQP1 may serve as a potential tumor suppressor gene in WT and could be a potential biomarker for the early diagnosis and prediction of disease progression in WT. In this study, we observed that overexpression of AQP1 suppressed the proliferation and promoted the apoptosis of WT cells. These findings were further validated in vivo. mRNA-Seq analysis of the AQP1 overexpression group and the control group revealed significant differential expression of STAT1, a key protein in the JAK-STAT signaling pathway, and caspase-1 and PYCARD (ASC), markers of cellular pyroptosis. Subsequent experimental validation confirmed that overexpression of AQP1 inhibited the JAK-STAT signaling pathway and activated the classic caspase-1-dependent pathway of cellular pyroptosis. We propose that the effects of AQP1 overexpression on cell proliferation and apoptosis in WT are mediated through the inhibition of the JAK-STAT signaling pathway and the promotion of caspase-1-dependent cellular pyroptosis. These findings provide new insights and potential strategies for the early diagnosis and treatment of WT involving AQP1.

There is limited research on the impact of AQP1 on cell proliferation. Previous studies have reported that during the late phase of renal ischemia/reperfusion (I/R) injury, both urinary exosomes and renal expression of AQP1 are significantly reduced. Concurrently, histological features of the kidney show increased tissue regeneration in segments associated with decreased AQP1 expression, accompanied by an increase in cell number and the presence of cells with a large nucleus. These findings suggest that the decrease in AQP1 expression may be associated with cell proliferation following renal I/R injury (Sonoda et al. 2009). Zhu et al. demonstrated that downregulation of AQP1 in mice with tibial fractures promotes the proliferation and differentiation of osteoblasts. This study suggested that the impact of AQP1 on osteoblast proliferation and differentiation is influenced by the p38 MAPK signaling pathway (Zhu et al. 2019). A recent study revealed that decreased expression of AQP1 located in the apical membranes of bile ducts in a gene-deficient mouse model results in increased proliferation of bile duct cells and periductal fibrosis. This study suggested that abnormal expression of AQP1 leads to alterations in intracellular ion transport systems, thereby causing a series of pathological changes associated with biliary diseases (Hatano et al. 2015). The exact mechanisms underlying the effect of AQP1 on tumor proliferation remain incompletely understood. Reports have shown that inhibition or overexpression of AQP1 has no significant impact on the proliferation of colon cancer cells and breast cancer cells (Dorward et al. 2016; Hu and Verkman 2006). However, in osteosarcoma cells, downregulation of AQP1 leads to inhibited tumor cell proliferation (Wu et al. 2015). In a recent study, Feng et al. demonstrated that AQP1 knockout (KO) mice exhibited increased growth of glioma cells, which was achieved through the MEK/ERK pathway. The use of MEK inhibitors significantly suppressed tumor growth (Hu et al. 2020). In this study, we observed that overexpression of AQP1 resulted in the inhibition of proliferation in WT cells. This finding was further confirmed through mRNA-Seq of the AQP1 overexpression group of WT cells, and Table 2 shows several significantly upregulated genes that are associated with tumor growth suppression. Furthermore, we conducted additional experiments to validate that overexpression of AQP1 led to the activation of STAT1 while inhibiting STAT3 and JAK2. Based on these findings, we proposed that the effect of AQP1 on proliferation may be mediated through the suppression of the JAK2/STAT3 signaling pathway. In summary, the overexpression of AQP1 may reduce the proliferation of WT cells by inhibiting the activation of JAK2/STAT3.

**Table 2:** KEGG pathways associated with the stable genes

KEGG pathways	*P* value	Genes
Influenza A	2.53E−14	Caspase-1, IFIH1, IRF7, PRSS3, OAS2, OAS1, RSAD2, OAS3, STAT1, MX2, CXCL10, MX1, CCL5, TLR3, DDX58, PYCARD
Hepatitis C	6.98E−10	IRF7, OAS2, OAS1, RSAD2, IFIT1, OAS3, STAT1, MX2, CXCL10, MX1, TLR3, DDX58
Measles	3.85E−08	IFIH1, IRF7, HSPA6, OAS2, OAS1, OAS3, STAT1, MX2, MX1, DDX58
NOD-like receptor signaling pathway	5.12E−08	Caspase-1, IRF7, OAS2, GBP4, OAS1, GBP5, OAS3, STAT1, GBP1, CCL5, PYCARD
Coronavirus disease—COVID-19	5.86E−08	Caspase-1, IFIH1, OAS2, ISG15, OAS1, OAS3, STAT1, MX2, CXCL10, MX1, TLR3, DDX58
Cytosolic DNA-sensing pathway	4.90E−06	Caspase-1, IRF7, CXCL10, CCL5, DDX58, PYCARD
Herpes simplex virus 1 infection	6.45E−06	IFIH1, IRF7, OAS2, SP100, ZNF562, OAS1, TAP1, OAS3, STAT1, CCL5, ZNF441, TLR3, DDX58, ZNF565
RIG-I-like receptor signaling pathway	9.11E−06	IFIH1, IRF7, ISG15, CXCL10, DHX58, DDX58
Epstein–Barr virus infection	1.08E−05	IRF7, OAS2, ISG15, OAS1, TAP1, OAS3, STAT1, CXCL10, DDX58
Toll-like receptor signaling pathway	8.75E−05	IRF7, STAT1, CXCL10, CCL5, CXCL11, TLR3
Human papillomavirus infection	0.00218751	ATP6V0A4, ISG15, AXIN2, STAT1, MX2, MX1, OASL, TLR3
Necroptosis	0.00525073	Caspase-1, STAT1, PYCARD, TLR3, JMJD7-PLA2G4B
Hepatitis B	0.00567976	IFIH1, IRF7, STAT1, TLR3, DDX58
Legionellosis	0.00758868	Caspase-1, HSPA6, PYCARD
Viral life cycle—HIV-1	0.00999516	MX2, MX1, SAMHD1

Plasma membrane AQP activity has been demonstrated to influence cellular apoptosis. Jablonski et al. (2004) showed that the inhibition of AQPs prevented apoptosis in granulocytes, thymocytes, and Chinese hamster ovary (CHO-AQP1) cells overexpressing AQP1. However, the overexpression of AQP1 promoted apoptosis. Importantly, these functional changes were not limited to specific cell types. The study suggested that AQPs primarily affected cell apoptosis through changes in cell volume, mitochondrial membrane potential, DNA degradation, and caspase-3 activation.

Recent studies have shown that the reduced expression levels of AQP1 in cardiomyocytes may decrease myocardial cell edema and apoptosis by regulating water transport and cell volume, thereby prolonging cellular viability (Huang et al. 2019). Decreased expression of AQP1 has also been found in brain cells and reduces cell apoptosis by inhibiting MAPK phosphorylation (Kim et al. 2015). In studies investigating the pathogenesis of osteoarthritis, researchers found that the expression of AQP1 is positively correlated with caspase-3. AQP1 activates caspase-3, thereby promoting apoptosis in chondrocytes (Gao et al. 2016). In our study, we also confirmed that overexpression of AQP1 promotes the expression and activation of caspase-3, which was consistent with the findings reported previously. We also validated the expression changes in the proapoptotic marker BAX. In conclusion, overexpression of AQP1 activated caspase-3, promoted the expression of BAX, and thereby induced apoptosis in WT cells.

Cell pyroptosis is a recently discovered form of programmed cell death characterized by the formation of protein pores on the cell membrane, cell swelling, and eventual rupture with the release of cellular contents (Al Mamun et al. 2021). Few studies have examined the role of AQPs in pyroptosis. Regarding the relationship between pyroptosis and tumors, increasing evidence suggests that pyroptosis plays an important role in tumors. However, the conclusions regarding the association between pyroptosis and tumors are not consistent.

Apoptosis inhibits tumor cell proliferation, and the acute inflammation induced by apoptosis enhances the immune response, suppressing tumor growth. Additionally, apoptosis can create a microenvironment conducive to tumor growth, thereby promoting tumor progression (Yu et al. 2021). In melanoma, it has been reported that the key effector molecule of pyroptosis, GSDME, exhibits tumor-suppressive effects. Melanoma cells lacking GSDME produced larger tumors than wild-type cells (Rogers et al. 2019). In contrast, another key molecule of pyroptosis, ASC, could activate IL-1β secretion and enhance NF-κB activity to promote the growth of metastatic melanoma. However, ASC overexpression in primary melanoma reduced the phosphorylation of IKKα/β, inhibiting NF-κB activity and exerting antitumor effects (Liu et al. 2013). In colorectal cancer, pyroptosis-associated inflammasomes inhibit tumorigenesis (Ma et al. 2018), and mice lacking ASC and caspase-1 are more prone to develop colorectal cancer (Dupaul-Chicoine et al. 2010). These findings provide further evidence for the antitumor effects of pyroptosis in colorectal cancer. In this study, we confirmed the significant impact of AQP1 overexpression on pyroptosis in WT cells. We observed a substantial increase in pyroptotic bodies in the AQP1 overexpression group. Additionally, key effectors of pyroptosis, including NLRP3, ASC, and GSDMD, were upregulated, while caspase-1 was activated, and IL-1β was increased. These results further support the notion that AQP1 overexpression may exert its effects through the activation of the caspase-1-dependent canonical pyroptotic pathway. The increased expression of ASC and activation of caspase-1 were consistent with our previous mRNA-Seq results. Moreover, given the impact of AQP1 overexpression on cell proliferation and apoptosis in WT found in prior studies, we proposed that pyroptosis may play a suppressive role in WT. Subsequently, we plan to further investigate the underlying mechanisms of the antitumor effects of pyroptosis in WT. In summary, AQP1 overexpression promoted pyroptosis in WT cells and then exerted antitumor effects in WT cells. This effect was achieved through the activation of the caspase-1-dependent canonical pyroptotic pathway.

## Conclusion

Overexpression of AQP1 suppressed the proliferation and promoted apoptosis of WT cells, which may be achieved through the inhibition of JAK2/STAT3 activation. Additionally, AQP1 overexpression facilitated pyroptosis in WT cells and exerted an antitumor effect. This effect was likely achieved through the activation of the caspase-1-dependent canonical pyroptotic pathway.

## Data Availability

All data generated or analyzed during this study are available in the GEO repository, GEO series accession number GSE247460.
